# Simultaneous Determination of Cortisol, Cortisone, and Multiple Illicit Drugs in Hair among Female Drug Addicts with LC-MS/MS

**DOI:** 10.3390/molecules26020516

**Published:** 2021-01-19

**Authors:** Cailing Duan, Yan Wu, Jin Yang, Shenghuo Chen, Yun Pu, Huihua Deng

**Affiliations:** 1 Key Laboratory of Child Development and Learning Science, Ministry of Education, Southeast University, Nanjing 210096, China; duan-cailing@seu.edu.cn (C.D.); rclswy@seu.edu.cn (Y.W.); yangjin@seu.edu.cn (J.Y.); chenshmessage@126.com (S.C.); 2 Institute of Child Development and Education, Research Center of Learning Science, Southeast University, Nanjing 210096, China; 3 Hangzhou College of Preschool Teacher Education, Zhejiang Normal University, Hangzhou 311231, China; 4 Women’s Compulsory Isolated Drug Rehabilitation Center, Nanjing 210031, China; hxcdcl@163.com

**Keywords:** hair matrix, cortisol, cortisone, illicit drugs, LC-MS/MS

## Abstract

Long-term dependence of illicit drugs impairs the function of the hypothalamic-pituitary-adrenal (HPA) axis, which regulates the secretion of endogenous steroids, cortisol, and cortisone. Thus, the present study aimed to develop a sensitive method for simultaneous determination of the multiple illicit drugs and two steroids in hair to monitor the status of illicit drug exposure and the physiological and psychological health of drug addicts. The target analytes were extracted from hair by incubation with 1 mL methanol for 24 h at 40 °C and then determined with LC-APCI^+^-MS/MS. The validated method showed acceptable linearity (*R*^2^ > 0.99) in the range of 1.25–250 pg/mg for cortisol and cortisone, 2.5–125 pg/mg for heroin, 2.5–1250 pg/mg for ketamine, 2.5–5000 pg/mg for methamphetamine (MAM), 2.5–250 pg/mg for 3, 4-methylenedioxymethamphetamine (MDMA), morphine, and 6-monoacetylmorphine (6-AM). Limits of quantification were 1.6, 1.2, 1.6, 1.0, 1.4, 0.3, 2.1, and 1.2 pg/mg for cortisol, cortisone, heroin, ketamine, MAM, MDMA, morphine, and 6-AM, respectively. Method recoveries were from 90–115% for all analytes. Inter-day and intra-day coefficients of variation were within 10%. Finally, this method was successfully applied to detect the aforementioned analytes in hair among female drug addicts who self-reported to be MAM abuser, heroin abuser, ketamine abuser, and abuser of mixture drugs of MAM and heroin. MAM abusers with current MAM use showed significantly higher concentrations of cortisol, MAM, and MDMA than controls with drug withdrawal.

## 1. Introduction

The illegal consumption of illicit drugs causes a series of serious physiological and psychological health problems [1,2]. MAM and MDMA, the prevalent drugs of abuse in China [3], have stimulant effects on central nervous system [4,5]. Ketamine induces the prolonged hallucination and delirium, which result in individual psychological dependence for continuous use [6]. As traditional drugs, heroin and its main metabolites, 6-AM and morphine are very dangerous in view of their high potential for dependence. Morphine is also an illicitly consumed drug. The presence of 6-AM is an indicator of the exposure to heroin [5,7]. Additionally, the concentration ratio of 6-AM to morphine reflects the purity of heroin being used [7,8]. Nowadays, the multiple illicit drugs can be potentially used by drug abusers [3,9]. As a consequence, an assay method for simultaneous identification and quantification of MAM, MDMA, ketamine, heroin, morphine, and 6-AM in bio-samples is required so as to reduce the amount of samples and the cost of analysis.

Bio-samples is required so as to reduce the amount of samples and the cost of analysis. Furthermore, the hypothalamic-pituitary-adrenal (HPA) axis plays a crucial role in drug addiction [10,11]. On the one hand, long-term dependence of illicit drugs damages the structure of the HPA axis or impairs its functions [12,13]. For example, heroin abuse gives rise to atypical circadian patterns of hormones’ secretion in the HPA axis and adrenal insufficiency [14]. Moreover, MDMA abuse is associated with the hyperactivity of the HPA axis [4,15,16]. On the other hand, HPA axis is usually activated in the exposure to chronic stress which plays a critical role in increasing additional vulnerability of drug abuse and risk of drug relapse [13].

Cortisol and cortisone as biomarkers of HPA axis to response to chronic stress can record the changes of HPA activity or stress exposure [17,18,19]. Therefore, simultaneous determination of cortisol, cortisone, and the aforementioned five illicit drugs and 6-AM will facilitate monitoring the status of drug exposure and the physiological and psychological health of drug abusers.

Traditionally, the bio-matrix used for the regular detection of illicit drugs is urine. While monitoring the detoxification and therapeutic processes for abusers of opiates and amphetamines, urinalysis possibly causes mistaken interpretation for complete abstinence from drugs abuse. It is due to the fact that the abused drugs in urine can only be detected within a few days after consumption [20]. Notably, hair analysis can eliminate the misinterpretation. Hair matrix with one centimeter to several centimeters in length might be a better biological matrix to record drug levels over one month to several months if hair growth rate is 1 cm per month [21,22]. In addition, hair holds some advantages of broader time-window of detection, non-invasive collection, high stability, and easy transportation and storage [23,24]. Considering the low concentrations of the eight analytes in human hair and the limited amount of hair sample available, an instrument with high sensitivity and selectivity is required to simultaneously determine of steroids and illicit drugs, while liquid chromatography-tandem mass spectrometry (LC-MS/MS) can satisfy the demand [3,7,25,26,27]. However, there has been no research reporting simultaneous determination of the two steroids, five illicit drugs, and 6-AM with LC-MS/MS at present.

In most previous studies, main ionization coupled to LC-MS/MS for determination of illicit drugs or 6-AM in hair was electrospray ionization (ESI) [2,3,24,26]. Unfortunately, none of these present studies can simultaneously detect of ketamine and heroin. Moreover, their limits of quantitation (LOQs) were too high (e.g., 200 pg/mg for MAM, MDMA, 6-AM and morphine) [7]. It might be attributed to the fact that the pulverized hair induced heavy matrix effect caused by co-eluted compounds when hair was incubated in methanol.

Considering that some target compounds (i.e., cortisol and cortisone) have low polarity or non-polarity, the derivatization to the molecules with higher polarity should be done ahead of the analysis when ESI in positive mode was used as ionization method. It is noticed that the derivatization produces gives rise to high background noise [17]. Thus, atmospheric pressure chemical ionization (APCI) is more appropriate than ESI for the ionization of cortisol and cortisone.

In brief, the present study firstly attempted to develop a sensitive LC-APCI^+^-MS/MS method for simultaneous determination of five illicit drugs (i.e., MAM, MDMA, ketamine, morphine, and heroin), 6-AM, and two glucocorticoids (i.e., cortisol and cortisone) in the non-pulverized hair from drug abusers. Furthermore, the assay would be applied to explore the washout effect, dosage effect, and withdrawal effect of illicit drug concentrations in five 1 cm hair segments from the scalp to the distal and the effect of drug withdrawal on the steroids’ levels among drug addicts.

## 2. Results

### 2.1. Chromatography

**Figure 1:**
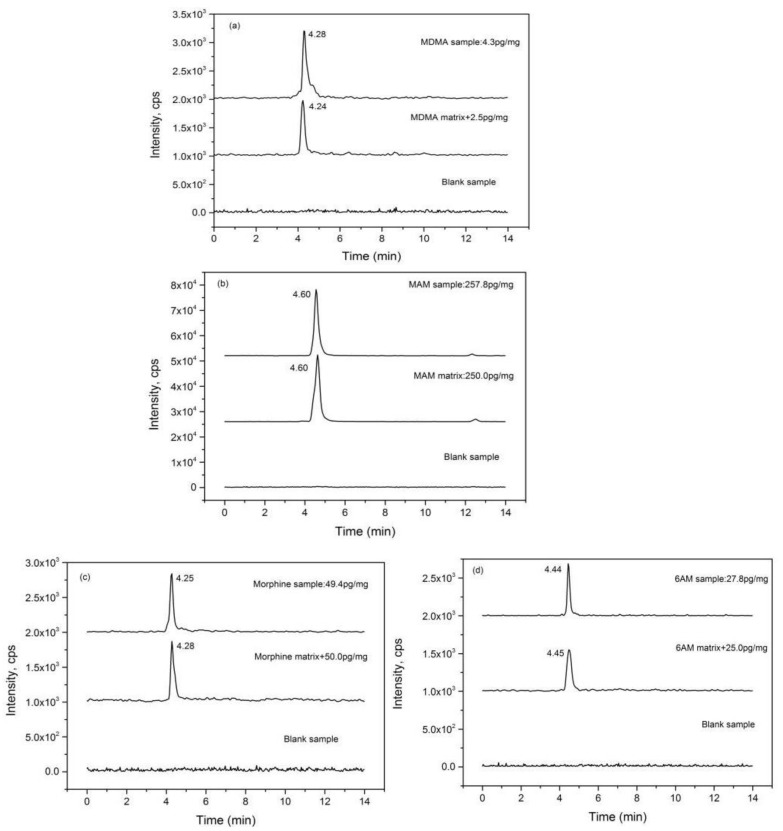

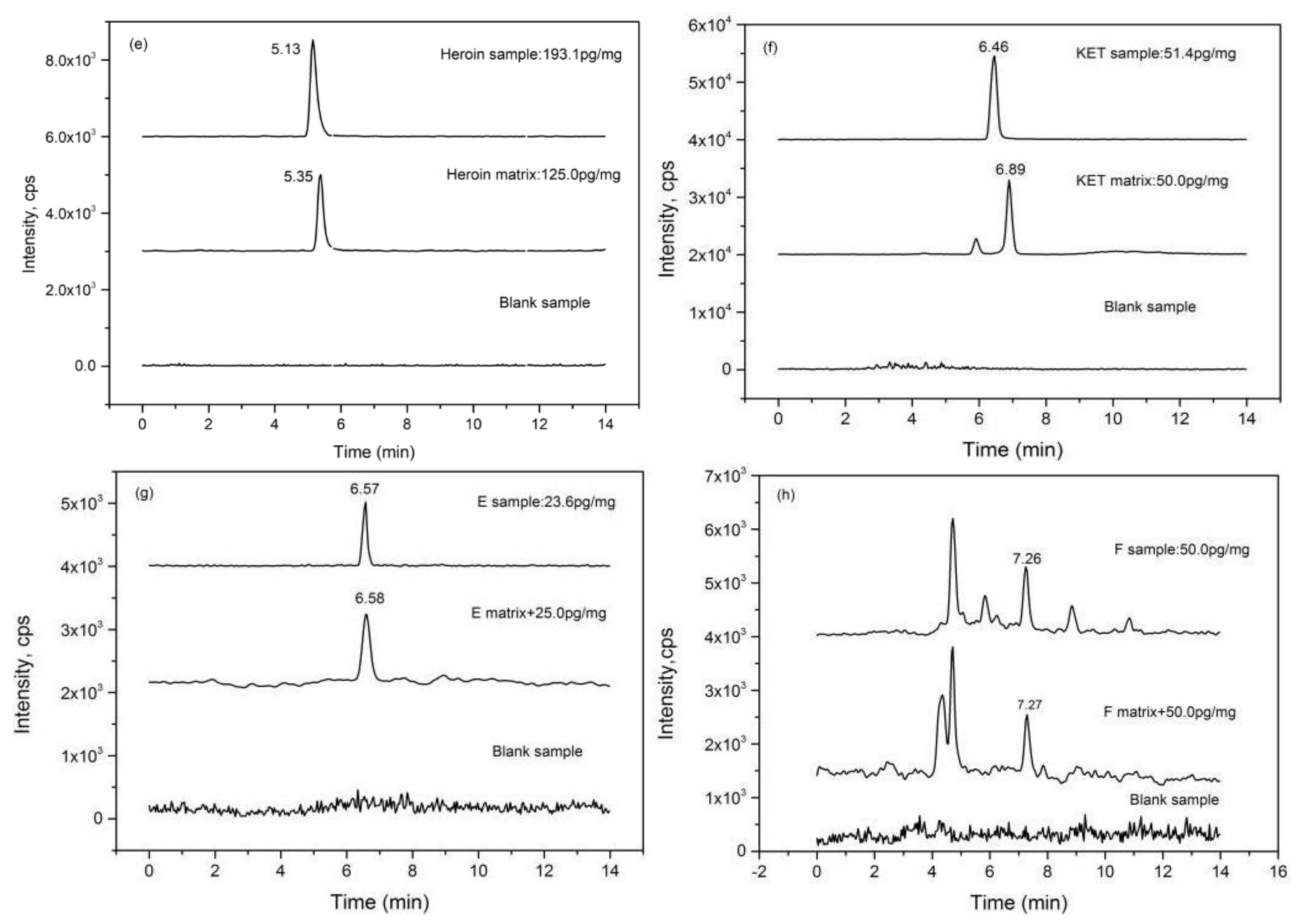
The chromatograms of blank hair matrix, the standard analytes spiked with blank hair matrix, and the analytes in natural hair samples. (**a**) MDMA, 3,4-methylenedioxymethamphetamine; (**b**) MAM, methamphetamine; (**c**) morphine, (**d**) 6-AM, 6-monoacetylmorphine; (**e**) heroin, (**f**) ketamine; (**g**) E, cortisone; and (**h**) F, cortisol.

As shown in Figure 1, MDMA, MAM, morphine, 6-AM, heroin, ketamine, cortisone, and cortisol were eluted at 4.24, 4.60, 4.28, 4.45, 5.35, 6.89, 6.58, and 7.27 min, respectively. Apparently, there were no other relevant peaks or interferences in the regions. These results indicated that the eight analytes could be well separated under the chromatographic conditions. In addition, low background noise from blank hair matrix showed that the present method had good selectivity and sensitivity for determination of the eight analytes in hair.

### 2.2. Validation

As listed in Table 1, the square of correlation coefficient of the calibration curve was more than 0.99 in the range of 2.5–5000 pg/mg for MAM, 2.5–250 pg/mg for MDMA, morphine and 6-AM, 2.5–1250 pg/mg for ketamine, 2.5–125 pg/mg for heroin, and 1.25–250 pg/mg for cortisol and cortisone, respectively. In addition, LOD and LOQ of each analyte were also summarized in Table 2.

As listed in Table 2, high recoveries were achieved at 90–115% for all analytes at five concentrations. Inter-day and intra-day coefficients of variation (CVs) were less than 10%. These results indicated that the present method had good performances in accuracy and precision. In addition, MAM, heroin, ketamine, and cortisol were affected by the matrix suppression, and morphine, MDMA, and cortisone had a lower matrix effect, which as shown in Figure S1 in the Supplemental Materials.

### 2.3. Analysis of Hair Samples

To further evaluate its applicability, the present LC-MS/MS method was used to simultaneously determine the concentrations of glucocorticoids, five illicit drugs, and 6-AM in five 1 cm hair segments from the scalp to the distal among 40 drug addicts. The typical results of MAM abusers, heroin abusers, ketamine abusers, and the mixed abuser of MAM and heroin were listed in Table 3. Among abusers who self-reported to use MAM (MAM abusers), the other five compounds (MDMA, ketamine, morphine, 6-AM, and even heroin) were identified in their hair strands where the concentration of MAM was higher than that of the rest. A similar pattern of polydrug abuse was observed for heroin abusers, ketamine abusers, and the mixed abuser of MAM and heroin. Additionally, heroin was identified in only several hair segments, while its concentrations in drug abusers were much lower compared to 6-AM and morphine. The hair concentrations of 6-AM and morphine in heroin users were much higher than those in most of abusers of the other drugs. The concentration ratios of 6-AM to morphine were less than 1.3 among the abusers except for S10.

### 2.4. The Washout Effect

**Figure 2:**
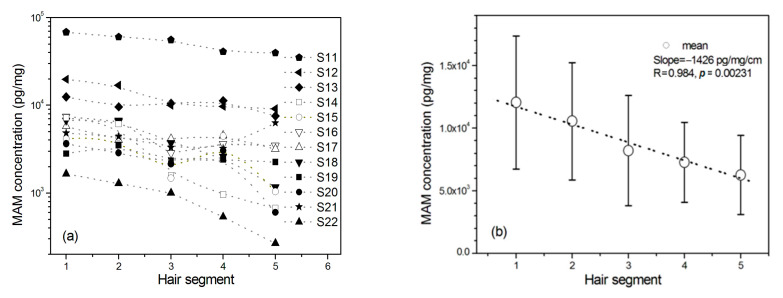
The changes of MAM concentration in 1 cm hair segment with the distance away from the scalp among MAM users whose dosages of MAM use were self-reported to be kept at the relatively stable amount per month in the past five months before they had been in drug rehabilitation center. Hair segment 1 is closest to the scalp. (**a**) MAM concentrations of 12 individuals (S11‒S22) and (**b**) mean MAM concentrations from 12 MAM abusers. Error bar in the figure is standard error mean.

As shown in Figure 2a, MAM concentrations in hair were decreased with the distance away from the scalp among 12 MAM abusers whose dosages of MAM use were self-reported to be kept at the relatively stable amount per month in the past five months (*n* = 12, *F*_(1.174, 12.916)_ = 5.279, *p* = 0.035, *η*^2^_p_ = 0.324). As shown in Figure 2b, the highest average concentrations were showed concentrations in the scalp-nearest segment among the five hair segments. The mean MAM concentrations were linearly dropped by 10.8% and with average linear rate at 1426 pg/mg per centimeter from the first segment to the next (r = 0.984, *p* < 0.01).

### 2.5. The Dosage Effect

**Figure 3:**
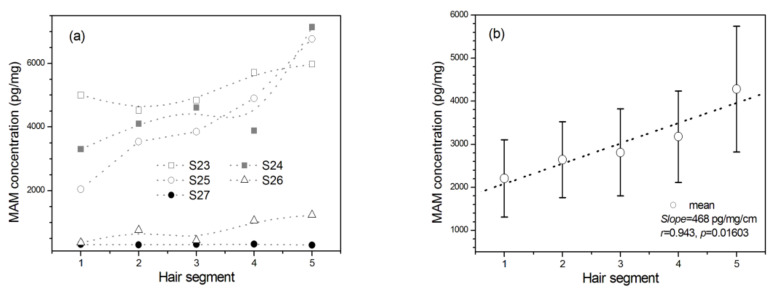
The changes of MAM concentration in 1 cm hair segment with the distance away from the scalp among MAM abusers whose amount of MAM use was self-reported to be gradually decreased in the past five months before they had been in drug rehabilitation center. Hair segment 1 is closest to the scalp. (**a**) MAM concentration of five individuals (S23‒S27) and (**b**) MAM mean concentrations from 5 MAM abusers. Error bar in the figure is standard error mean.

As shown in Figure 3a, MAM concentrations were increased with the distance away from the scalp among 5 MAM abusers whose amount of MAM use was self-reported to be gradually decreased in the past five months (*n* = 5, *F*_(4, 16)_ = 4.514, *p* = 0.012, *η*^2^_p_ = 0.530). As shown in Figure 3b, it showed the highest average concentrations in the fifth segment among the five hair segments. The mean MAM concentrations were linearly increased by 29.0% and with average linear rate at 468 pg/mg per centimeter from the one segment to the next (*r* = 0.943, *p* < 0.05).

### 2.6. The Withdrawal Effect

**Figure 4:**
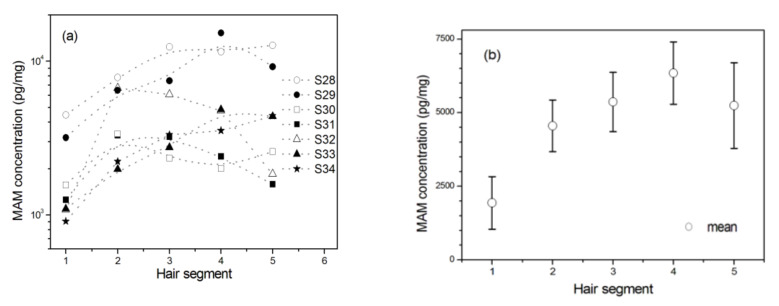
The changes of MAM concentration in 1 cm hair segment with the distance away from the scalp among MAM abusers who were prohibited to use MAM in the past one month before they had been in drug rehabilitation center. Hair segment 1 is closest to the scalp. (**a**) MAM concentrations of seven individuals (S28‒S34) and (**b**) MAM mean concentrations from seven MAM users. Error bar in the figure is standard error mean.

As shown in Figure 4a, there were significant differences in MAM concentrations among five hair segments for seven MAM abusers who were prohibited to use MAM in the past one month (*n* = 7, *F*_(4, 24)_ = 4.618, *p* = 0.007, *η*^2^_p_ = 0.435). However, there were no significant differences among the second, third, fourth, and fifth segments (*n* = 7, *F*_(3, 18)_ = 0.907, *p* = 0.457, *η*^2^_p_ = 0.131). Post hoc multiple comparisons revealed that MAM concentrations in the scalp-nearest segment were significantly lower than those in the other four segments (*p*s < 0.05). As shown in Figure 4b, they showed the lowest average levels in the scalp-nearest segment among the five hair segments.

**Figure 5:**
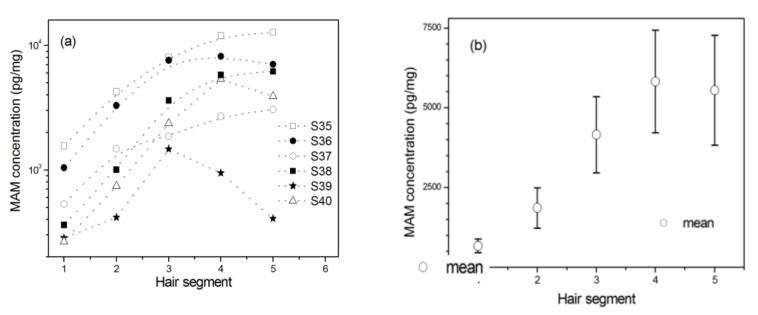
The changes of MAM concentration in 1 cm hair segment with the distance away from the scalp among MAM abusers who were prohibited to use MAM in the past two months before they had been in drug rehabilitation center. Hair segment 1 is closest to the scalp. (**a**) MAM concentrations of six individuals (S35‒S40) and (**b**) MAM mean concentrations from six MAM users. Error bar in the figure is standard error mean.

**Figure 6:**
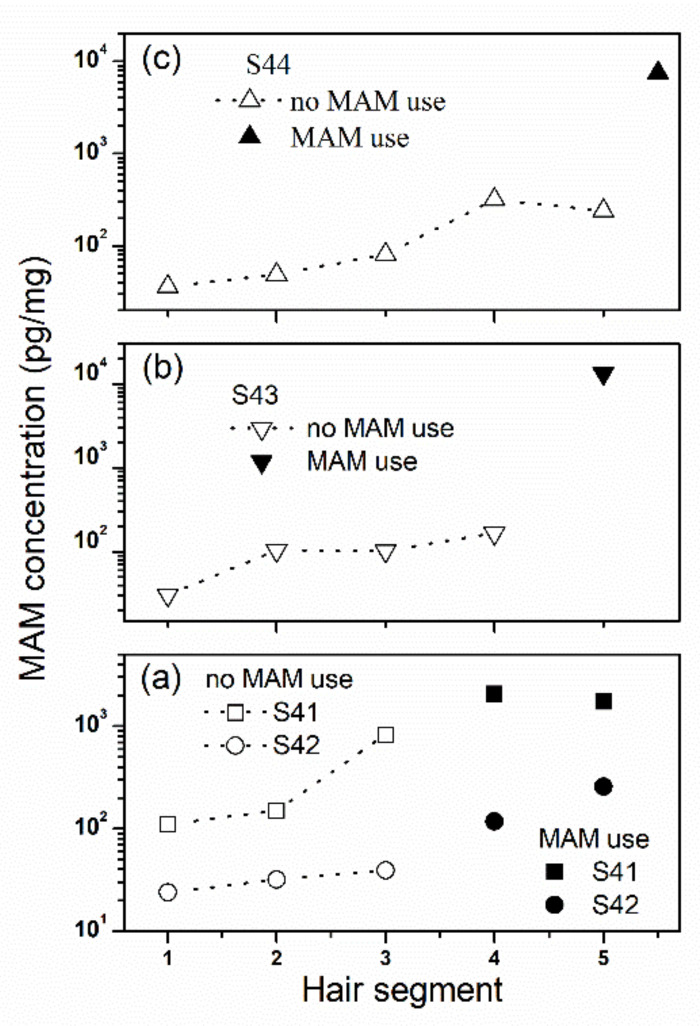
The changes of MAM concentration in 1 cm hair segment with the distance away from the scalp among MAM abusers who were prohibited to use MAM in (**a**) the past three months, (**b**) the past four months, and (**c**) the past five months before they had been in drug rehabilitation center. Hair segment 1 is closest to the scalp. (**a**) S41 and S42, and (**b**) S43 and (**c**) S44.

For MAM abusers who were prohibited to use MAM in the past two months, MAM concentrations showed significant differences among five hair segments (*n* = 6, *F*_(1_._282, 6_._408)_ = 10.334, *p* = 0.014, *η*^2^_p_ = 0.674), while there were no significant differences among the third, fourth, and fifth segments (*n* = 6, *F*_(2, 10)_ = 3.602, *p* = 0.066, *η*^2^_p_ = 0.419) as shown in Figure 5a. Post hoc multiple comparisons revealed that MAM concentrations in the scalp-nearest segment were significantly lower than those in the other four segments (*p*s < 0.05) and those in the second segment were also significantly lower than those in the third, fourth, and fifth segments (*p*s < 0.05). As shown in Figure 5b, they showed the lowest average levels in the scalp-nearest segment among five hair segments. Similar withdrawal effects were observed for MAM abusers who were prohibited to use MAM in the past three, four, and five months as shown in Figure 6. Notably, the concentrations of MAM in hair remained detectable levels even if MAM abusers had withdrawal from MAM for five months.

### 2.7. The Effect of MAM Use on Concentrations of Steroids in Hair

**Figure 7:**
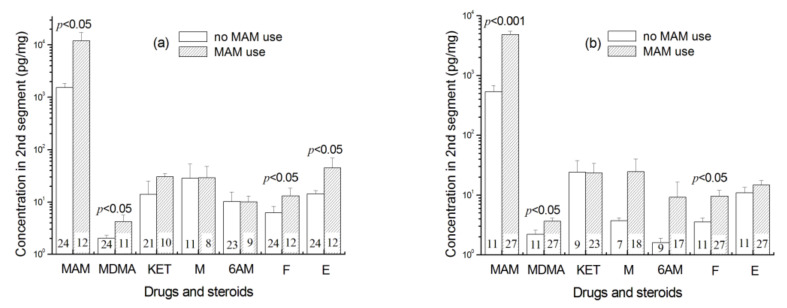
Comparison between MAM users and controls in the concentrations of MAM, MDMA, ketamine, morphine (M), 6-AM, cortisol (F), and cortisone (E) in (**a**) the first hair segment and (**b**) the second hair segment. (**a**) Twelve MAM abusers vs. 24 controls with MAM withdrawal, and (**b**) 27 MAM abusers vs. 11 controls with MAM withdrawal. Several participants were excluded because their concentrations of ketamine, morphine, 6-AM, and heroin were lower than the limits of detection. Error bar in the figure is standard error mean.

The *t*-test for two independent samples revealed that relative to the controls with MAM withdrawal, MAM users with current MAM use showed significantly higher concentrations of MAM, MDMA, cortisol, and cortisone in the first 1 cm segment (12,000 ± 18,391 vs. 1307 ± 1333 pg/mg, *t*_34_ = 2.388, *p* = 0.027 for MAM, and 4.2 ± 4.6 vs. 1.5 ± 0.8 pg/mg, *t*_33_ = 2.335, *p* = 0.028 for MDMA, and 12.9 ± 10.3 vs. 4.7 ± 3.3 pg/mg, *t*_34_ = 2.212, *p* = 0.038 for cortisol, and 44.9 ± 44.5 vs. 12.4 ± 9.5 pg/mg, *t*_34_ = 2.816, *p* = 0.012 for cortisone) and significantly higher concentrations of MAM, MDMA and cortisol in the second 1 cm segment (4822 ± 3611 vs. 533 ± 500 pg/mg, *t*_28_._343_ = 6.029, *p* < 0.001 for MAM; 3.6 ± 2.3 vs. 2.2 ± 1.3 pg/mg, *t*_32_._655_ = 2.413, *p* = 0.022 for MDMA; 9.5 ± 13.8 vs. 3.5 ± 2.1 pg/mg, *t*_28_._754_ = 2.189, *p* = 0.037 for cortisol) as shown in Figure 7. However, there were no significant differences between them in ketamine, morphine, and 6-AM in the first 1 cm segment (*p*s > 0.342) and in ketamine, morphine, 6-AM, and cortisone in the second 1 cm segment (*p*s > 0.418).

## 3. Discussion

Glucocorticoids (i.e., cortisol and cortisone), illicit drugs (i.e., MAM, MDMA, ketamine, morphine and heroin), and 6-AM in natural hair samples from drug abusers were successfully and simultaneously quantified with the present LC-APCI^+^-MS/MS method. To our best knowledge, this is the first successful attempt to simultaneously determine the eight compounds in hair under APCI in positive mode.

Its applicability was also confirmed by significantly higher hair cortisol concentrations in MAM abusers with current MAM use relative to the controls with drug withdrawal. The present result was consistent with previous results that psychoactive drugs (e.g., MAM and MDMA) induced higher concentrations of cortisol in saliva and hair [4,15,16]. However, the present MAM users also used other illicit drugs, such as heroin or morphine as demonstrated in Section 2.3 and Section 2.7. Heroin and morphine are believed to be associated with suppressing the basal activity of the HPA axis [12,14]. Therefore, the present MAM abusers with current MAM use showed the hyperactivity of the HPA axis might imply that MAM plays a more important impact on the HPA activity than heroin and morphine. This might also result from the use of much higher MAM dosage (Figure 7) although they used other illicit drugs, such as heroin or morphine.

The present method showed LOD and LOQ at 0.7 and 1.6 pg/mg for heroin, respectively. To our knowledge, this is the first successful attempt to get the detectable concentration of heroin from several hair samples of drug abusers using LC-APCI-MS/MS. This is mainly because the heroin content in human hair is really low for its fast degradation in blood [28]. However, previous studies were not able to detect heroin from natural hair when they incubated with acetonitrile [26] and phosphates buffer [24]. One reason for the discrepancy might be that the extraction of heroin from the pulverized hair produced heavy matrix effect, resulting in high LOD and LOQ, such as 10 and 50 pg/mg for LOD and LOQ. The other reason might be long-term sample preparation (i.e., liquid-liquid extraction and solid-phase extraction [26]), which leads to the hydrolysis from heroin to 6-AM and from 6-AM to morphine.

It was worthy to point out that the present positive result was not enough to exclude the possible contamination of heroin from the atmosphere. When drug addicts used heroin with the smoking manner [7], heroin in the atmosphere might enter into the outer layer and even the inner layer of three-layer hair structure. Heroin in the outer layer of the three-layer hair might be completely removed because hair was frequently washed with shampoo solution and hot water in daily life [21,22]. It was demonstrated by the fact that heroin could not be detected in the washing solvent after the 3-min cleaning with methanol done in this study. In contrast, the heroin in the inner layer of hair structure could not be removed by daily hair-washing and a 3-min cleaning with methanol. Because heroin that injected into blood could be fast degraded, the detectable concentration of hair heroin implied the potential use of the smoking manner or the combination of smoking with other manner, such as injection.

The present study showed LOD and LOQ matched or lower than those in previous publications for cortisol, cortisone, MAM, MDMA, ketamine, morphine and 6-AM. For instance, Chen et al. [17] and Christopher et al. [29] reported LODs at 2 and 0.5 pg/mg for cortisol and cortisone. Lendoiro et al. [26] reported LODs at 2 pg/mg for ketamine, MAM, MDMA and 6-AM, and at 5 pg/mg for morphine. Imbert et al. [24] obtained LODs at 5 pg/mg and LOQs at 50 pg/mg for both MAM and MDMA, and LODs at 5 and 20 pg/mg for 6-AM and morphine, and LOQs at 50 pg/mg for both 6-AM and morphine. Klys et al. [7] reported LOQs at 200 pg/mg for MAM, MDMA, 6-AM, and morphine. The achievement of the good performances in the present method might be attributed to the improvements as follows. Firstly, previous studies detected illicit drugs with LC-ESI-MS/MS. LC-ESI-MS/MS may be more susceptible to matrix effect than LC-APCI-MS/MS, especially for the present analytes with weak polarity [30,31]. It is because APCI is based on gas-phase reactions and ESI is mainly on liquid-phase reactions [32]. Secondly, previous studies extracted analytes with aqueous acids [33] or buffer solutions [24] and acetonitrile [26] rather than methanol. These extraction solutions have stronger polarity and penetration ability than methanol. Therefore, the solutions might dissolve more other compounds out of hair matrix, resulting in the heavier matrix effect. For instance, Domínguez-Romero et al. [33] used aqueous solutions, such as acidic and alkaline solutions (0.1 M HCl and 0.5 M NaOH) to extract illicit drugs from hair. The basic substances (e.g., opiates and amphetamines) in acidic conditions can be effectively extracted by aqueous HCl due to the protonation of the nitrogen atom(s) presenting in the molecules and a solubility increase in aqueous solutions after the protonation. However, there was a hydrolysis of hair matrix (e.g., catalysis by NaOH under alkaline conditions) and dissolution of a lot of other compounds, which resulted in heavy matrix effect. Additionally, Lendoiro et al. [26] used acetonitrile for the extraction of MAM, MDMA, ketamine, morphine, and 6-AM in hair. Although better extraction yielded and cleaner extracts were obtained, the cleanup procedure was rather onerous. The hair samples were incubated in acetonitrile for 12 h at 50 °C and then purified through liquid-liquid extraction and solid-phase extraction. Thus, methanol might be a better reagent for extracting both illicit drugs and steroids in hair relative to acetonitrile and aqueous solutions. Thirdly, previous studies combined other methods in the pretreatment of hair samples (i.e., pulverizing hair or ultra-sonication) [7,26] resulting in heavy matrix effect, especially for steroids. Klys et al. [7] used 20mg of pulverized hair in combination with ultra-sonication for extracting MAM, MDMA, morphine, and 6-AM. Although APCI was used, the LOQs were still achieved at 200 pg/mg, which were worse than the present results. Methanol extraction coupled with ultra-sonication has been proven to be better choice for extracting illicit drugs where methanol penetrates the hair matrix, thereby leading to hair’s swelling and drug liberation via diffusion. Besides, this process could be fastened by ultra-sonication that caused a strong degradation of hair structure [22]. However, the determinations of endogenous cortisol and cortisone are easily affected by other impurities produced by ultra-sonication because their contents are much lower than those of exogenous illicit drugs in hair. Therefore, the present LC-APCI^+^-MS/MS method based on the extraction from 20 mg non-pulverized hair with methanol was a more sensitive and simpler method that might be more adaptive to simultaneously determine the two glucocorticoids, five illicit drugs, and 6-AM. In addition, the results of the above limits of quantification and the method recoveries (from 90–115% for all analytes) in the present study are better than the achievements in a Chinese related research “Study on standard method for detection of toxic substances in human biological samples (blood and hair)” [34].

The present study further found that MAM abusers had the pattern of polydrug abuse although they self-reported to mainly use MAM (Table 4). The pattern of polydrug abuse was also observed for ketamine abuser, heroin abuser, and a mixed abuser of MAM and heroin. The observation was in accordance with the results in the study of Klys et al. [7] where drug addicts often take opiates together with amphetamine and/or its derivatives (i.e., MAM and MDMA). Additionally, the present study found that most of heroin addicts showed very low and even undetectable concentrations of hair heroin, and showed much higher concentrations of hair morphine and 6-AM than most of abusers using other drugs (Table 4). This might attributed to the fact that heroin is rapidly metabolized to morphine via 6-AM, such as the fast degradation in blood. 6-AM is usually considered as the proof of heroin use. The present finding implied that heroin abuse could be verified through the identification of hair 6-AM with the present method, and the manner of heroin use (i.e., smoking) could be verified through the identification of hair 6-AM and heroin as mentioned above. Notably, the ratios of 6-AM to morphine were less than 1.3 in hair from most of the present abusers except for S10, indicating that they might use heroin with high impurity [7].

## 4. Materials and Methods

### 4.1. Participants

The participants were female drug addicts recruited from a women’s compulsory isolated drug rehabilitation center in Nanjing, Jiangsu Province, China. All participants provided written informed consent prior to inclusion. The present study followed the Declaration of Helsinki and was approved by the Health Science Research Ethics Board of Southeast University.

The included participants were older than 18 years and had history of drug dependence that met the DSM-IV criteria more than one year. In contrast, the exclusion criteria were: (1) Body mass index (BMI) > 28 kg/m^2^; (2) mental disorders including post-trauma stress disorder; (3) HIV infection; (4) chronic diseases (i.e., hepatic, renal, cardiovascular, and pulmonary diseases); (5) medication including the use of glucocorticoid drugs or antidepressants over the past 5 months; (6) dyed, permed, bleached, or short hair (<5 cm).

The participants initially included 145 females. Then, three drug addicts were excluded due to HIV medication or mental disorders. Five addicts were excluded because of BMI > 28 kg/m^2^, 11 addicts for medication, and 86 addicts for dyed, perm, bleached, or short hair (<5 cm). Finally, 40 drug addicts remained in the study. They self-reported the information on the types and dosages of drugs used over last five months and the duration in the drug rehabilitation center. Moreover, they also provided hair samples longer than 5 cm that possibly record the true status of drug abuse over the past 5 months. Of them, 34 addicts self-reported to be MAM abusers before they entered the drug rehabilitation center, and 4 addicts were heroin abusers, 1 addict, was a mixed abuser of heroin and MAM, and 1 addict was ketamine abuser. They had no chances to use any illicit drugs once they entered the drug rehabilitation center. Among them, 13 MAM addicts had been in the drug rehabilitation center for one month and 11 MAM addicts for two months. Then, they did not use MAM for one month and two months, respectively.

### 4.2. Chemicals and Solutions Preparation

Standard solutions of MAM, MDMA, ketamine, morphine, 6-AM, heroin, and the deuterated compounds, methamphetamine-D5 as internal standards (IS) of MAM and MDMA, ketamine-D4 as IS of ketamine, and morphine-D3 as IS of morphine, 6-AM, and heroin were purchased from Beijing Bafang Century Technology Ltd., Beijing, China. The purity of the above compounds is over 96%. Methanol, cortisol, cortisone, and their ISs, cortisol-D4 and cortisone-D7, were obtained from Sigma Aldrich, St. Louis, MO, USA. Acetic acid and ammonium acetate were from Tedia, Fairfield, OH, USA. All solvents were of HPLC grade. Water used throughout the experiments was triple-distilled deionized water.

Stock solutions of standards, cortisol, cortisone, MAM, MDMA, morphine, 6-AM, and heroin were prepared in methanol at the concentrations of 1 mg/mL and ketamine at 100 µg/mL. Methamphetamine-D5, morphine-D3, ketamine-D4, cortisol-D4, and cortisone-D7 were also prepared at 100 µg/mL in methanol. Working solutions of drug standards were finely diluted with methanol to the desired concentrations (e.g., 0.5, 1.0, 2.0, 5.0, 10.0, 20.0, 50.0, 100.0, 200.0, 500.0, 1000.0, and 2000.0 ng/mL for MAM, MDMA, ketamine, heroin 6-AM, and morphine; 0.1, 0.2, 0.5, 1.0, 2.0, 5.0, 10.0, 20.0, 50.0, 100.0, and 200.0 ng/mL for cortisol and cortisone). All the internal standard mixtures were obtained in methanol at the final concentration of 20 ng/mL. All solutions were stored at −20 °C prior to use.

### 4.3. Extraction of Analytes in Hair

Hair strands longer than 5 cm in the posterior vertex were cut with scissors as close as possible to the scalp. Then, they were cut in five 1 cm hair segments from the nearest-scalp segment to the hair distal for experimental use.

Before incubation in methanol, 1 cm hair samples were finely cut into pieces of 1–2 mm in length using perfectly decontaminated scissors. The 20 mg samples were introduced into a 2 mL clean and dry tube and washed for one minute with 1 mL methanol for a total of three times. Then they were dried at room temperature immediately. Sequentially, 950 µL of methanol and 50 µL of 20 ng/mL IS mixture were added. The resulting samples were incubated at 40 °C for 24 h. After incubation, hair samples were vortex-mixed for 5 s and centrifuged at 12,000 rpm for 5 min. Then 800 µL supernatant was transferred to a new 2 mL dry tube and evaporated under nitrogen flux at 50 °C. Finally, the dry residue was re-dissolved with 50 µL of mobile phase for the next LC-APCI^+^-MS/MS analysis.

### 4.4. Simultaneous Analysis of the Eight Analytes in Hair

Chromatographic separation of the analytes was achieved on Agilent 1200 HPLC system (Agilent Technologies, Waldbronn, Germany). The 20 µL of the re-dissolved solution was injected into a Platisil ODS-C18 analytical column (5 µm, 150 mm × 4.6 mm; Dikma Technologies, Beijing, China), which was protected by a C18 guard cartridge (5 µm, 10 mm × 4.6 mm; Dikma Technologies, Beijing, China). The mobile phase consisted of a mixture of methanol and deionized water (80:20, *v/v*) with 2 mM ammonium acetate (pH = 4.5). It was filtered through the micro porous membrane (0.22 µm) prior to use. The column temperature was set at 40 ± 1 °C and the flow rate was 400 µL/min. As a result, the total run time was 15 min.

Detection was performed on a 3200 QTRAP tandem mass spectrometer (AB Sciex, Foster City, CA, USA) controlled by the Analyst^®^ 1.5 software. The instrument was operated with an atmospheric pressure chemical ionization (APCI) in positive mode to produce protonated molecules of analytes under following optimized settings: The symmetrical heaters at 450 °C, nebulizer current at 4.0 µA, collision gas at Medium, curtain gas at 20.0 psig, gas 1 (ion source gas) at 40 psig, and gas 2 at 40.0 psig,. High-purity nitrogen (99.999%) was utilized for nebulizing gas. Data were acquired in multiple reactions monitoring mode. The precursor ion and product ion of each target compounds in the optimum condition and its chemical structure were shown in Figure S2 (details seen in the Supplemental Materials). The conditions for ionization and fragmentation were optimized for eight analytes as listed in Table 4. All analytes were monitored with a dwell time of 120 ms.

### 4.5. Method Validation

The present method was validated in standards spiked with 20 mg hair blank matrix that was 1 cm segment in the hair distal 27–31 cm away from the scalp of a drug-free female adult. It was due to the effect that the analytes’ contents in the hair segment were below the limits of detection of this method under the washout effect and solarizing effect [35]. Calibration curves were prepared at considered ranges (i.e., 1–2000 ng/mL for illicit drugs and 0.1–200.0 ng/mL for cortisol and cortisone). The concentration of each standard solution was used as x-coordinate of calibration curve and the peak area ratio of standard to IS was used as y-coordinate.

The intra-day and inter-day precisions and analytical recovery were evaluated at five concentrations (i.e., 2.5, 250, 500, 1250, and 5000 pg/mg for MAM, MDMA, and ketamine, 5, 250, 500, 1250, and 5000 pg/mg for morphine, 6-AM, and heroin, 2.5, 25, 50, 125, 500 pg/mg for cortisone and cortisol). Recovery was estimated by the ratio of each analyte’s content calculated from calibration curve to the standard amount. Limit of detection (LOD) was defined as the lowest concentration with acceptable chromatography where the presence of all transitions with the signal-to-noise ratio (*S*/*N*) at 3. Similarly, limit of quantitation (LOQ) was the lowest concentration, which can be identified with *S*/*N* at 10.

### 4.6. Statistical Methods

Statistical analysis was conducted with SPSS 18.0 for windows. Repeated measure analysis of variance (ANOVA) with Greenhouse–Geisser correction was conducted for comparison of hair drugs and steroids among multiple hair segments and post hoc multiple comparisons based on least significant difference was conducted for comparison between any two segments. *t*-test for two independent samples was conducted for comparison of hair drugs and steroids between MAM abusers and controls without MAM use.

## 5. Conclusions

An assay for simultaneous determination of cortisol, cortisone, MAM, MDMA, ketamine, morphine, heroin, and 6-AM in hair was successfully developed based on LC-APCI+-MS/MS. All analytes were extracted from 20 mg non-pulverized hair with 1 mL methanol incubation. The present method achieved good performances, especially for LOD and LOQ at 0.7 and 1.6 pg/mg for cortisol, 0.5 and 1.2 pg/mg for cortisone, 0.6 and 1.4 pg/mg for MAM, 0.1 and 0.3 pg/mg for MDMA, 0.4 and 1.0 pg/mg for ketamine, 0.9 and 2.1 pg/mg for morphine, 0.7 and 1.6 pg/mg for heroin, and 0.5 and 1.2 pg/mg for 6-AM, and method recoveries were from 90 to 115% for all analytes. The above results in the present study are better than the achievements in a Chinese-related research “Study on standard method for detection of toxic substances in human biological samples (blood and hair)” [35]. Its applicability was confirmed by the successful detection of the concentrations of the eight analytes in hair from female drug addicts who self-reported to use different illicit drugs. Its applicability was also confirmed by the result that MAM abusers with current MAM use showed significantly higher hair cortisol concentrations than controls with the drug withdrawal. The present LC-APCI-MS/MS protocol was a sensitive and simple method being more adaptive to simultaneously monitor both the status of drug exposure and the physiological and psychological health of drug abusers. However, our research only identified five illicit drugs, one metabolite, and two steroids, which needed to be further validated under the stricter conditions for more kinds of illicit drugs, metabolites, and steroids.

Additionally, this study found that there was a washout effect, dosage effect, and withdrawal effect that influences MAM concentrations in hair from MAM abusers. The results indicated that the information on hair washing, drug dosage, and drug withdrawal should be considered when the concentrations of illicit drugs in hair are applied to monitor the status of long-term drug exposure of drug abusers.

## Figures and Tables

**Table 1:** Linearity, square of correlation coefficient (R2), calibration range, limit of detection (LOD), and limit of quantitation (LOQ) results for the eight compounds.

Compound	Calibration Curve	*R* ^2^	Linear Range (pg/mg)	LOD (pg/mg)	LOQ (pg/mg)
MAM	y = 0.0217x + 0.0028	0.9997	2.5–5000	0.6	1.4
MDMA	y = 0.0416x + 0.0062	0.9996	2.5–250	0.1	0.3
Ketamine	y = 0.0258x + 0.0059	0.9998	2.5–1250	0.4	1.0
Morphine	y = 0.0189x + 0.0036	0.9971	2.5–250	0.9	2.1
6-AM	y = 0.0321x + 0.0061	0.9979	2.5–250	0.5	1.2
Heroin	y = 0.023x + 0.0052	0.9998	2.5–125	0.7	1.6
E	y = 0.3967x + 0.0155	0.9997	1.25–250	0.5	1.2
F	y = 0.0264x + 0.0077	0.9906	1.25–250	0.7	1.6

Notes: MAM, methamphetamine; MDMA, 3,4-methylenedioxymethamphetamine; 6-AM, 6-monoacetylmorphine; E, cortisone; F, cortisol.

**Table 2:** Intra-day and inter-day coefficients of variation (CVs) and method recovery for the eight compounds.

Compound	Nominal (pg/mg)	Intra-Day CV (%, *n* = 5)	Inter-Day CV (%, *n* = 5)	Recovery (%, *n* = 5)
	2.5	5.2	3.8	97.5 ± 5.1
	250	2.1	2.9	102.6 ± 0.1
MAM	500	1.7	1.3	101.6 ± 1.7
	1250	1.5	1.4	99.9 ± 1.5
	5000	1.7	2.7	100.4 ± 1.7
	2.5	4.6	4.4	99.2 ± 4.6
	250	1.9	1.9	100.8 ± 1.9
MDMA	500	1.6	2.4	102.7 ± 1.7
	1250	2.9	4.2	107.6 ± 3.1
	5000	2.2	2.9	106.5 ± 2.3
	2.5	3.7	3.5	102.2 ± 3.7
	250	1.4	1.8	101.7 ± 1.4
Ketamine	500	1.8	2.6	102.0 ± 1.9
	1250	1.3	1.9	100.8 ± 1.3
	5000	2.8	4.1	106.1 ± 0.8
	5	3.5	4.2	104.3 ± 3.6
	250	3.5	3.0	102.8 ± 3.6
Morphine	500	2.7	2.8	105.5 ± 2.9
	1250	2.5	2.2	100.4 ± 2.6
	5000	2.0	5.4	104.3 ± 2.0
	5	9.4	5.6	104.6 ± 9.9
	250	2.3	4.3	102.5 ± 2.3
6-AM	500	3.3	4.4	106.5 ± 3.5
	1250	2.8	2.8	100.9 ± 2.8
	5000	3.6	3.8	100.6 ± 3.6
	5	5.9	4.7	106.3 ± 6.3
	250	1.3	3.4	100.5 ± 1.3
Heroin	500	1.4	2.7	94.9 ± 1.4
	1250	1.4	1.8	96.5 ± 1.3
	5000	1.6	1.7	96.4 ± 1.6
	2.5	2.0	3.8	103.7 ± 2.1
	25	5.9	3.2	100.3 ± 5.9
E	50	3.0	2.2	101.0 ± 3.0
	125	5.1	2.4	97.0 ± 4.9
	500	3.2	1.6	106.8 ± 3.4
	2.5	5.1	4.9	100.3 ± 5.9
	25	1.9	3.3	103.8 ± 1.9
F	50	5.4	5.4	104.5 ± 5.6
	125	1.4	2.5	101.1 ± 1.4
	500	3.2	4.0	103.0 ± 3.3

Notes: MAM, methamphetamine; MDMA, 3,4-methylenedioxymethamphetamine; 6AM, 6-monoacetylmorphine; E, cortisone; F, cortisol.

**Table 3:** The concentrations of MAM, MDMA, ketamine, morphine, 6-AM, heroin, cortisone, cortisol, and the ratio of 6-AM to morphine in five 1 cm hair segments from 10 female addicts.

Drugs Used from Self-Report	Segment ^c^	MAM	MDMA	Ketamine	Morphine	6-AM	Heroin	E	F	Ratio ^d^
MAM	S01-1	6,710.2	2.1	43.6	158.9	12.5	—	23.6	1.9	0.08
	S01-2	6,736.1	2.0	66.0	96.0	2.9	—	9.5	1.7	0.03
	S01-3	3,715.8	1.9	29.0	125.8	1.7	—	3.3	3.3	0.01
	S01-4	2,608.8	184.2	165.5	316.0	224.6	192.0	5.2	15.2	0.7
	S01-5	1,173.9	1.0	12.0	238.2	—	—	4.2	3.0	—
MAM	S02-1	1,081.0	2.2	142.6	—	17.4	—	9.0	7.3	—
	S02-2	6,692.1	3.6	10.9	—	—	—	14.3	8.9	—
	S02-3	6,061.2	2.7	10.6	2.3	0.7 ^e^	—	2.1	3.4	0.3
	S02-4	4,746.4	137.4	135.7	—	—	4.6	12.7	2.1	—
	S02-5	1,848.6	1.0	15.6	—	—	—	4.4	2.7	—
MAM	S03-1	12,467.0	1.6	12.2	10.2	8.6	—	7.2	10.1	0.8
	S03-2	9,613.0	8.9	18.0	7.4	—	—	9.7	11.0	—
	S03-3	10,521.7	8.0	26.8	4.3	1.4	—	6.0	5.5	0.3
	S03-4	11,312.7	108.9	145.3	3.0	4.8	11.7	15.9	11.0	1.6
	S03-5	7,538.1	2.8	57.2	7.3	—	—	4.4	2.5	—
MAM	S04-1	2,052.2	1.1	4.2	—	5.1	—	5.6	8.0	—
	S04-2	3,539.2	3.6	1.1	1.6	—	—	7.2	4.7	—
	S04-3	3,851.1	4.1	3.2	9.7	2.5	3.7	5.6	4.0	0.3
	S04-4	4,902.3	0.8	1.0	2.6	—	—	9.8	2.7	—
	S04-5	6,768.7	3.8	1.6	4.9	—	—	9.0	8.3	—
Heroin	S05-1	342.8	15.2	8.7	750.4	492.5	—	15.8	4.3	0.7
	S05-2	181.6	9.2	174.0	631.3	415.4	—	4.5	4.5	0.7
	S05-3	208.9	11.4	10.6	876.4	628.4	—	8.3	2.1	0.7
	S05-4	107.4	10.2	1.5	596.0	467.1	—	3.8	—	0.8
	S05-5	81.1	9.9	7.1	693.6	522.9	2.0	6.0	—	0.8
Heroin	S06-1	117.0	2.2	5.1	10.5	9.3	—	7.7	10.6	0.9
	S06-2	309.8	2.5	1.3	45.8	31.2	—	4.8	3.4	0.7
	S06-3	1,517.7	4.5	7.6	213.5	83.8	—	5.9	10.0	0.4
	S06-4	2,712.5	3.7	5.3	420.1	147.0	—	6.5	12.7	0.3
	S06-5	3,323.4	3.4	8.9	905.6	216.6	1.3 ^e^	4.7	3.9	0.2
Heroin	S07-1	77.0	5.3	6.9	671.0	270.7	2.6	17.4	10.1	0.4
	S07-2	239.3	8.1	10.1	724.2	363.2	1.4 ^e^	9.1	—	0.5
	S07-3	322.0	10.5	50.9	673.2	327.9	1.3 ^e^	9.9	2.5	0.5
	S07-4	312.3	11.8	38.9	881.6	434.6	—	8.8	8.4	0.5
	S07-5	683.2	7.6	129.0	818.5	344.0	3.5	6.7	10.9	0.4
Heroin	S08-1	21.4	6.3	—	7.6	2.0	1.7	6.6	1.8	0.3
	S08-2	24.0	10.2	—	17.6	1.8	—	13.7	11.1	0.1
	S08-3	47.3	9.2	1.5	5.0	1.4	—	4.2	14.6	0.3
	S08-4	19.1	4.2	1.0	19.3	9.5	—	7.8	3.3	0.5
	S08-5	15.6	6.0	3.1	122.8	77.9	—	5.5	2.4	0.6
Mixture ^a^	S09-1	24,090.8	11.2	97.1	3.6	—	—	19.0	2.3	—
	S09-2	30,000.2	10.6	188.7	2.2	1.2	—	8.2	10.7	0.5
	S09-3	26,015.9	13.3	108.4	2.8	1.3	—	43.2	9.4	0.5
	S09-4	39,305.9	11.6	10.1	3.6	1.6	—	9.1	5.0	0.4
	S09-5	42,462.0	10.7	29.8	2.5	1.6	—	19.8	7.1	0.6
Ketamine ^b^	S10-3	306.3	8.4	14,543.2	5.0	23.2	—	7.2	6.5	4.6
	S10-4	619.9	8.5	21,827.6	2.3	22.5	—	11.6	1.8	9.8
	S10-5	461.3	8.8	24,738.6	2.3	—	—	7.7	—	—

Notes: MAM, methamphetamine; MDMA, 3,4-methylenedioxymethamphetamine; 6-AM, 6-monoacetylmorphine; E, cortisone; F, cortisol. The symbol “—” meant the concentration below the limit of detection. ^a^ It refers to the mixed use of MAM and heroin. ^b^ The first and second hair segments of the participant were excluded because they were contaminated in the analysis. ^c^ Four MAM female abusers were S01‒S04, 4 heroin abusers were S05‒S08, 1 abuser of mixed drug was S09, and 1 ketamine abuser was S10. Hair segment 1 was the scalp-nearest 1 cm segment, and segment 2–5 were the 1 cm segment 2–5 cm away from the scalp, respectively. ^d^ It refers to the ratio of 6-AM to morphine. ^e^ The values were less than LOQ, but more than LOD. They were evaluated by the calibrated curves.

**Table 4:** Optimum ionization and fragmentation conditions for cortisol, cortisone, six drugs, and their internal standards.

Analytes	Q1/Q3 (m/z)	DP(V)	EP(V)	CEP(V)	CE(V)	CXP(V)
MAM	150.1/118.9 *	22.72	6.63	12.71	14.53	2.35
	150.1/90.6	22.72	6.63	12.71	21.60	3.05
MAM-D5	155.2/120.9	28.96	2.81	10.81	14.07	2.17
MDMA	194.1/163.2 *	28.15	3.54	17.22	16.42	2.79
	194.1/135.1	28.15	3.54	17.22	32.13	2.41
Heroin	370.2/165.2 *	53.29	5.36	35.64	71.49	3.52
	370.2/152.0	53.29	5.36	35.64	95.49	4.20
6-AM	328.1/165.2 *	51.29	3.05	34.84	50.90	2.14
	328.1/211.3	51.29	3.05	34.84	35.34	2.13
Morphine	286.4/152.1 *	52.43	3.46	30.84	71.46	80.66
	286.4/127.9	52.43	3.46	30.84	1.61	2.85
Morphine-D3	289.2/152.0	52.88	5.19	38.91	74.37	1.65
Ketamine	238.2/125.1 *	35.11	4.62	34.05	38.77	2.47
	238.2/179.2	35.11	4.62	34.05	25.04	4.17
Ketamine-D4	242.3/129.1	35.24	4.28	34.58	37.97	3.34
Cortisol	363.1/121.1 *	43.87	5.04	24.20	31.35	2.88
Cortisol-D4	367.1/121.1 *	40.00	5.04	24.39	37.00	3.50
Cortisone	361.1/163.1 *	53.94	5.83	24.11	31.11	2.85
Cortisone-D7	368.2/169.0 *	48.00	5.83	24.44	32.00	3.50

Notes: MAM, methamphetamine; MDMA, 3, 4-methylenedioxymethamphetamine; 6-AM, 6-monoacetylmorphine; E, cortisone; F, cortisol; DP, declustering potential; EP, entrance potential; CEP, collision cell entrance potential; CE, collision energy; CXP, collision cell exit potential. For each species, the most sensitive transition (marked as *) was used for quantitation (quantifier) and the second one was used for confirmation (qualifier).

## Data Availability

Data available on request due to restrictions e.g., privacy and ethical. The data presented in this study are available on request from the corresponding author.

## Supplementary Materials

The following are available online at. Figure S1: The effect of hair matrix on the sensitivity for cortisol, cortisone, methamphetamine, 3,4-methylenedioxymethamphetamine, ketamine, heroin, 6-monoacetylmorphine, morphine; Figure S2: The precursor ion and product ion of each target compounds in the optimum condition.

## Sample Availability

Samples of the compounds, cortisol, cortisone, heroin, morphine, 6-monoacetylmorphine, ketamine, methamphetamine and 3, 4-methylenedioxymethamphetamine, are available from the authors.
