# Supplementary material for: Simultaneous Determination of Cortisol, Cortisone, and Multiple Illicit Drugs in Hair among Female Drug Addicts with LC-MS/MS
Source: Molecules. 2021 Jan 19;26(2):516. doi: 10.3390/molecules26020516 (PMC7835904; doi:10.3390/molecules26020516)
Supplement: Supplementary file 1 [file molecules-26-00516-s001.pdf]

molecules

MDPI

Supplementary Materials

Simultaneous Determination of Cortisol, Cortisone, and Multiple Illicit Drugs in Hair among Female Drug Addicts with LC-MS/MS

Cailing Duan [1,2], Yan Wu [1], Jin Yang [1], Shenghuo Chen [1], Yun Pu [3] and Huihua Deng [1,*]

1 Research Center for Learning Science, Key Laboratory of Child Development and Learning Science (Southeast University), Ministry of Education, and Institute of Child Development and Education, Southeast University, Nanjing 210096, China; duan-cailing@seu.edu.cn (C.D.); rclswy@seu.edu.cn (Y.W.); yangjin@seu.edu.cn (J.Y.); chenshmessage@126.com (S.C.)
2 Hangzhou College of Preschool Teacher Education, Zhejiang Normal University, Hangzhou 311231, China
3 Women's Compulsory Isolated Drug Rehabilitation Center, Nanjing 210031, China; hxcdcl@163.com
* Correspondence: dengrcls@seu.edu.cn; Tel.: +86-25-83795664; Fax: +86-25-83793779

1. The effect of hair matrix on the sensitivity

The matrix effect of our internal standard method was assessed by coefficients of variation in the sensitivity (CVs) of the calibration curves where CVs = (S_{matrix}-S)/S×100% with S_{matrix} and S represented the sensitivities with and without blank matrix, respectively. The sensitivity was defined as the instrument response of 1 pg/mg analytes and could be calculated as the slope in the fitted linear dependence of the calibration curve. According to the formula, we achieved the vales of the CVS are -15.4% for MAM, -10.7 % for ketamine, -2.2 % for morphine, -12.7 %for heroin, -17.3 % for cortisol, 5.4 % for MDMA, 14.2 % for 6-AM, 2.6 % for cortisone, which were shown as Figure S1. A negative value indicates matrix suppression while a positive value indicates matrix enhancement. Obviously, methamphetamine, heroin, ketamine and cortisol are affected by the matrix suppression. The value of CVS was less than 10%, such as morphine, MDMA, and cortisone, which indicates the less matrix effect.

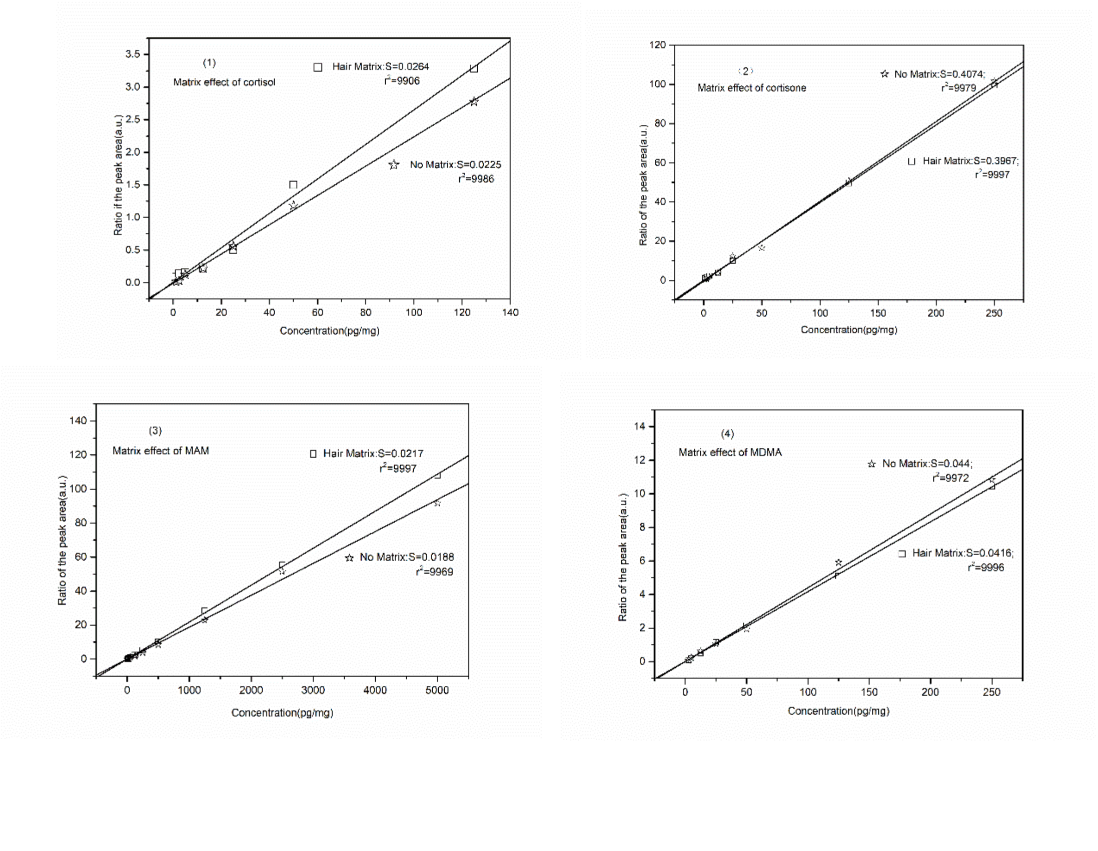

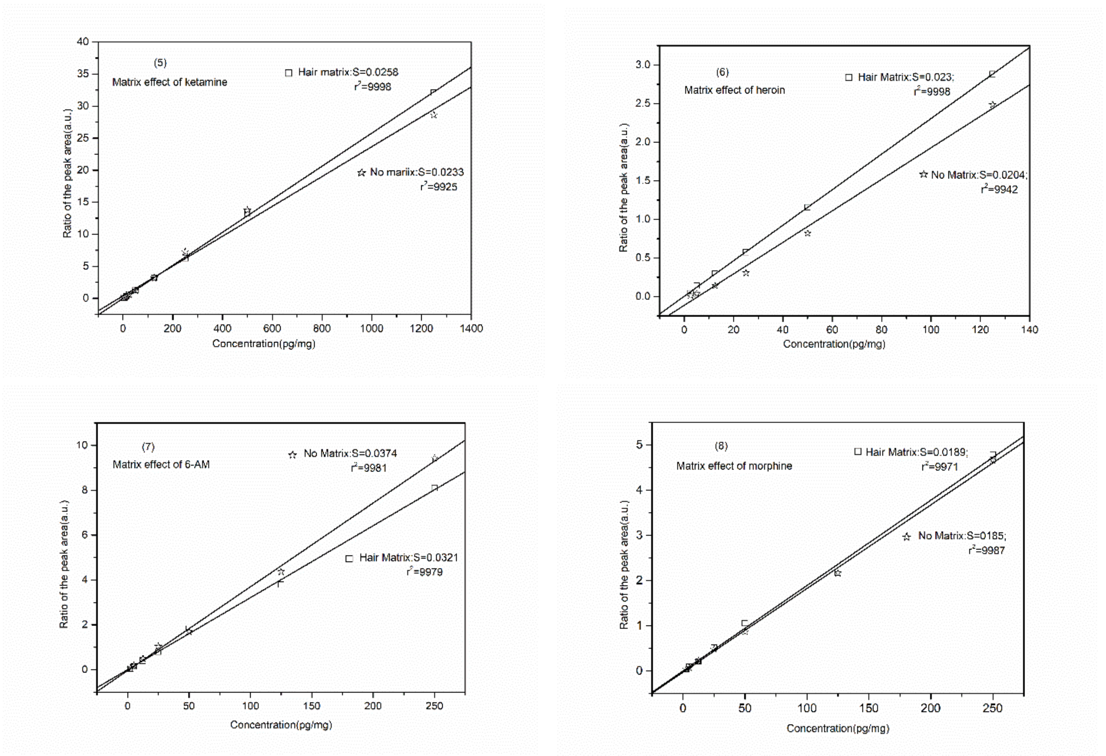

Figure S1. The effect of hair matrix on the sensitivity. (1) cortisol, (2) cortisone, (3) MAM, methamphetamine, (4) MDMA, 3,4-methylenedioxymethamphetamine; (5) ketamine, (6) heroin, (7) 6-AM, 6-monoacetylmorphine; (8) morphine.

2. The precursor ion and product ion of each target compounds in the optimum condition

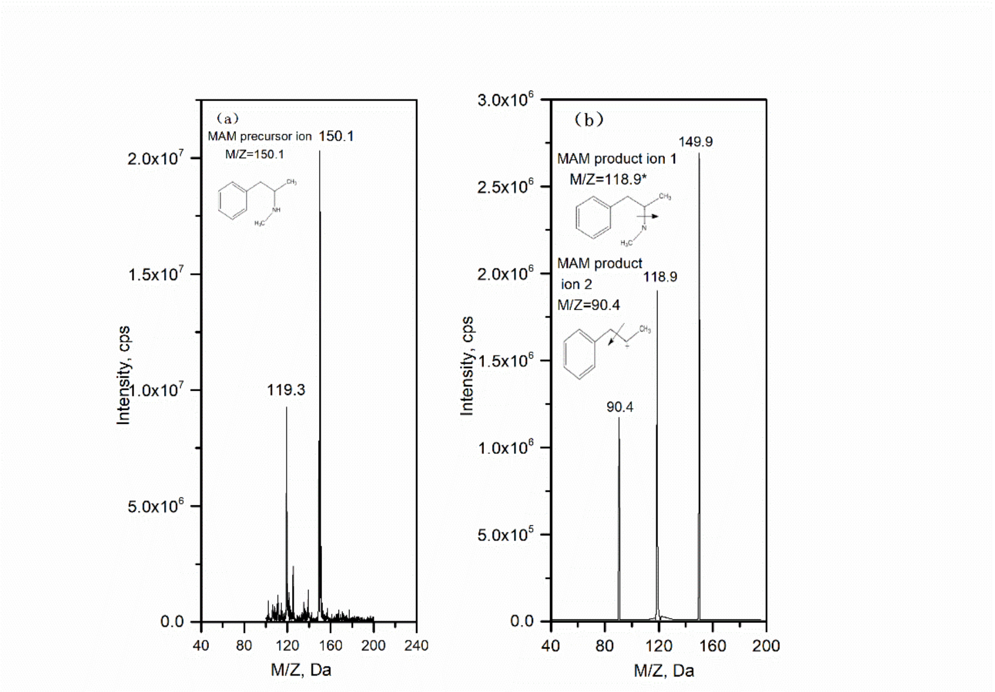

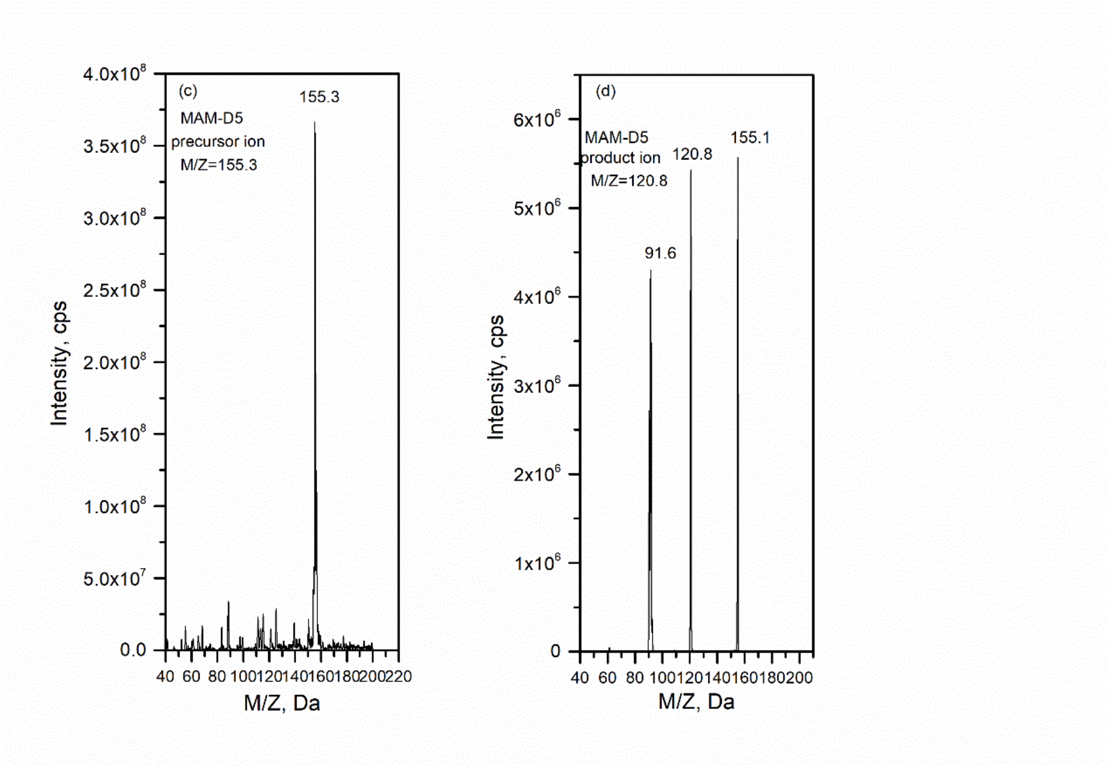
(c)
MAM-D5
precursor ion
M/Z=155.3
155.3
Intensity, cps
M/Z, Da
(d)
MAM-D5
product ion
M/Z=120.8
91.6
120.8
155.1
Intensity, cps
M/Z, Da

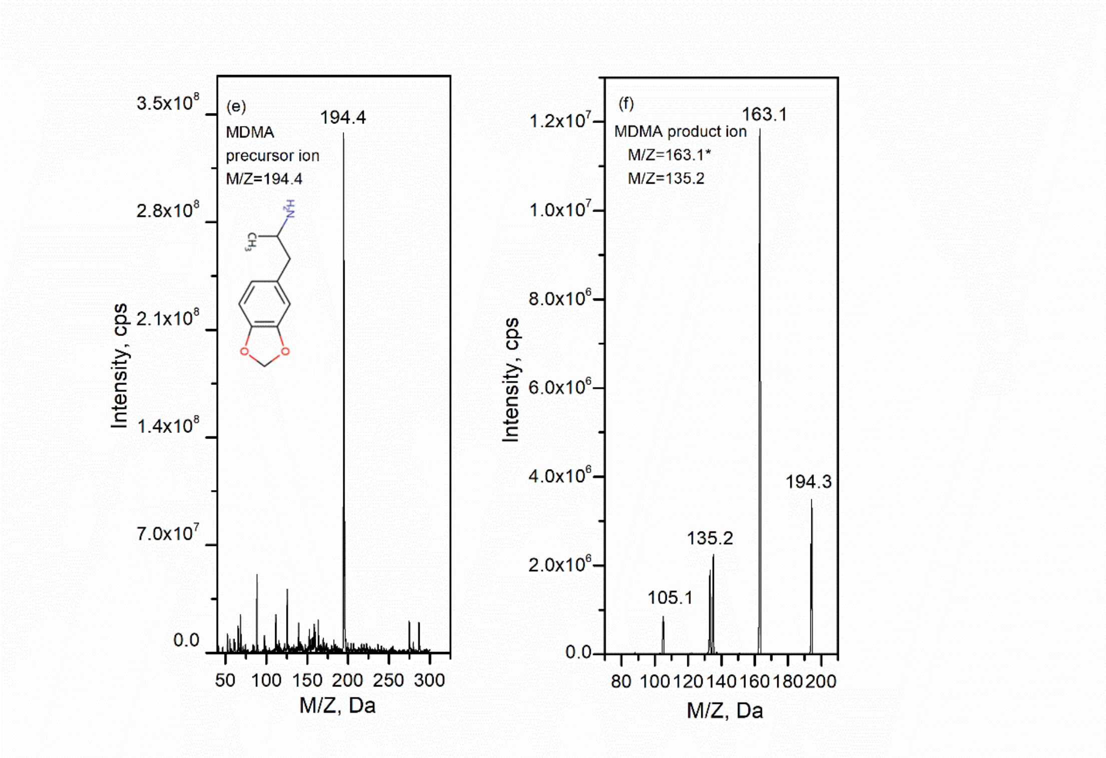
(e)
MDMA
precursor ion
M/Z=194.4
194.4
Intensity, cps
M/Z, Da
(f)
MDMA product ion
M/Z=163.1*
M/Z=135.2
163.1
194.3
135.2
105.1
Intensity, cps
M/Z, Da

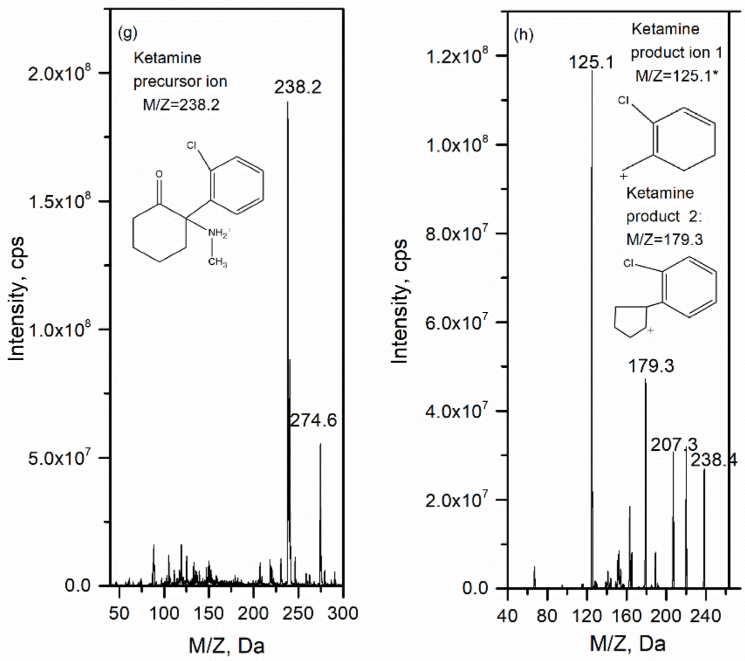
(g)
Ketamine
precursor ion
M/Z=238.2
238.2
274.6
Intensity, cps
M/Z, Da
(h)
Ketamine
product ion 1
M/Z=125.1*
Ketamine
product 2:
M/Z=179.3
125.1
179.3
207.3
238.4
Intensity, cps
M/Z, Da

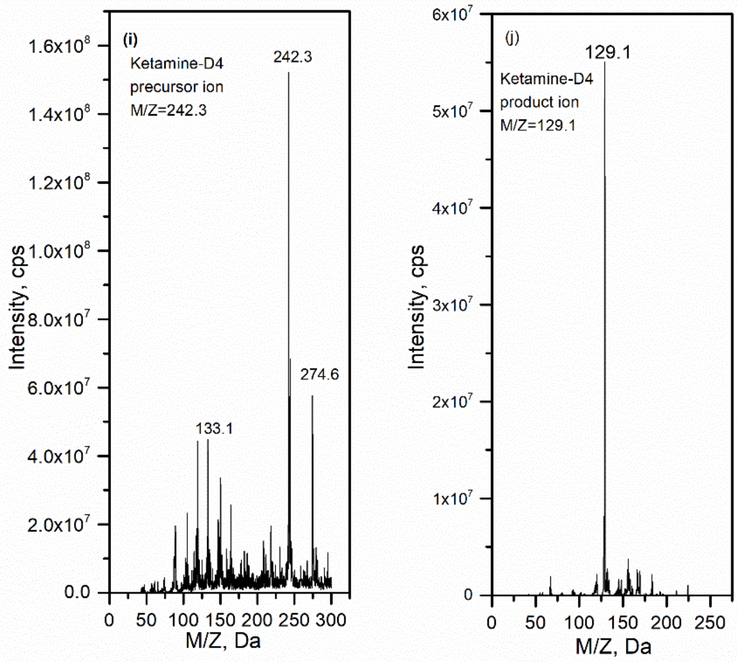
(i)
Ketamine-D4
precursor ion
M/Z=242.3
242.3
274.6
133.1
Intensity, cps
M/Z, Da
(j)
Ketamine-D4
product ion
M/Z=129.1
129.1
Intensity, cps
M/Z, Da

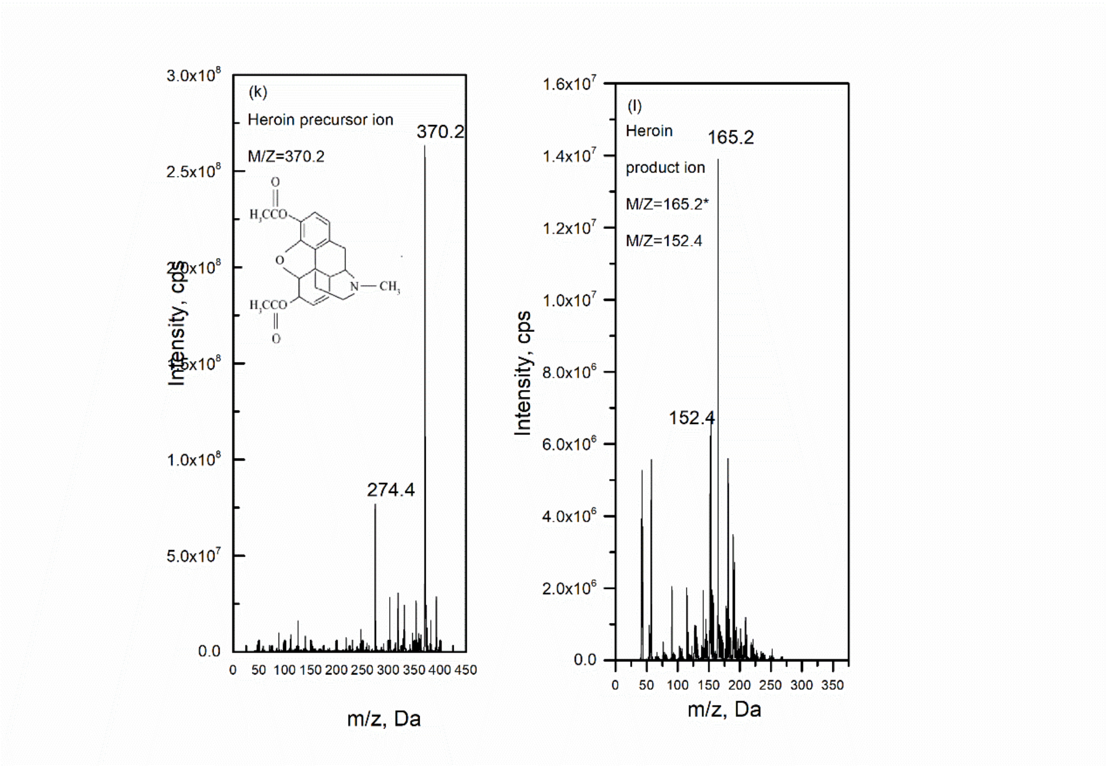
(k)
Heroin precursor ion
M/Z=370.2
370.2
274.4
Intensity, cps
m/z, Da
(l)
Heroin
product ion
M/Z=165.2*
M/Z=152.4
165.2
152.4
Intensity, cps
m/z, Da

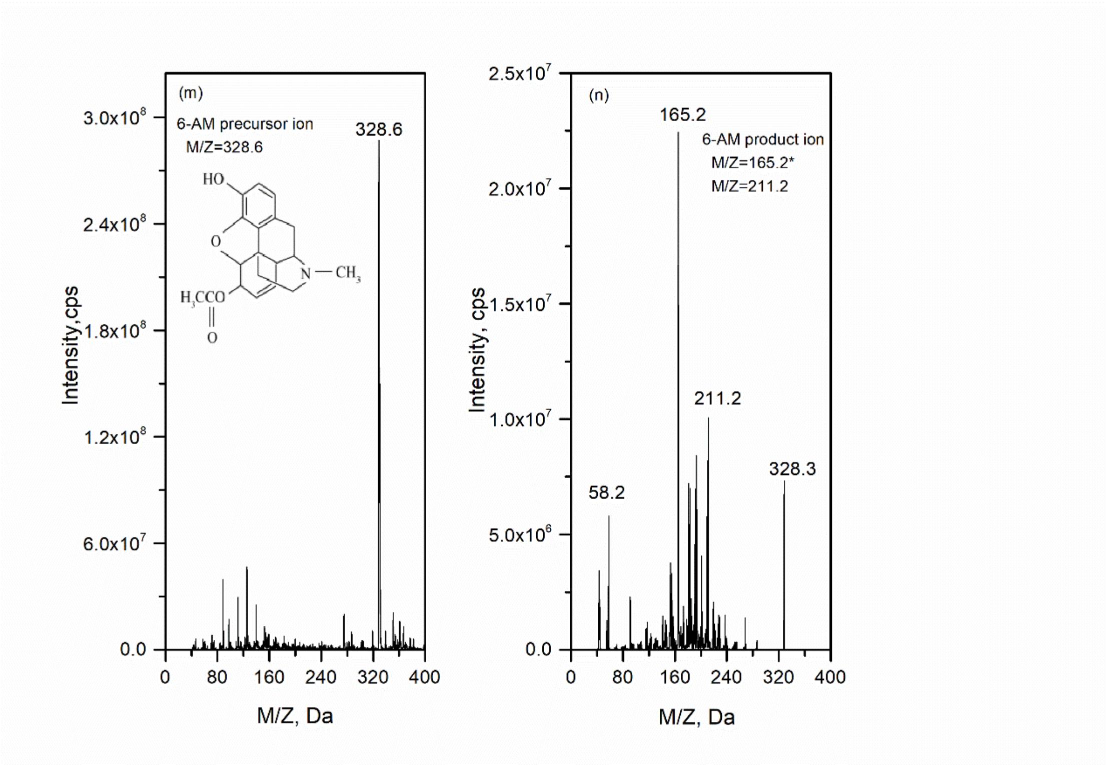
(m)
6-AM precursor ion
M/Z=328.6
328.6
Intensity,cps
M/Z, Da
(n)
165.2
6-AM product ion
M/Z=165.2*
M/Z=211.2
211.2
328.3
58.2
Intensity, cps
M/Z, Da

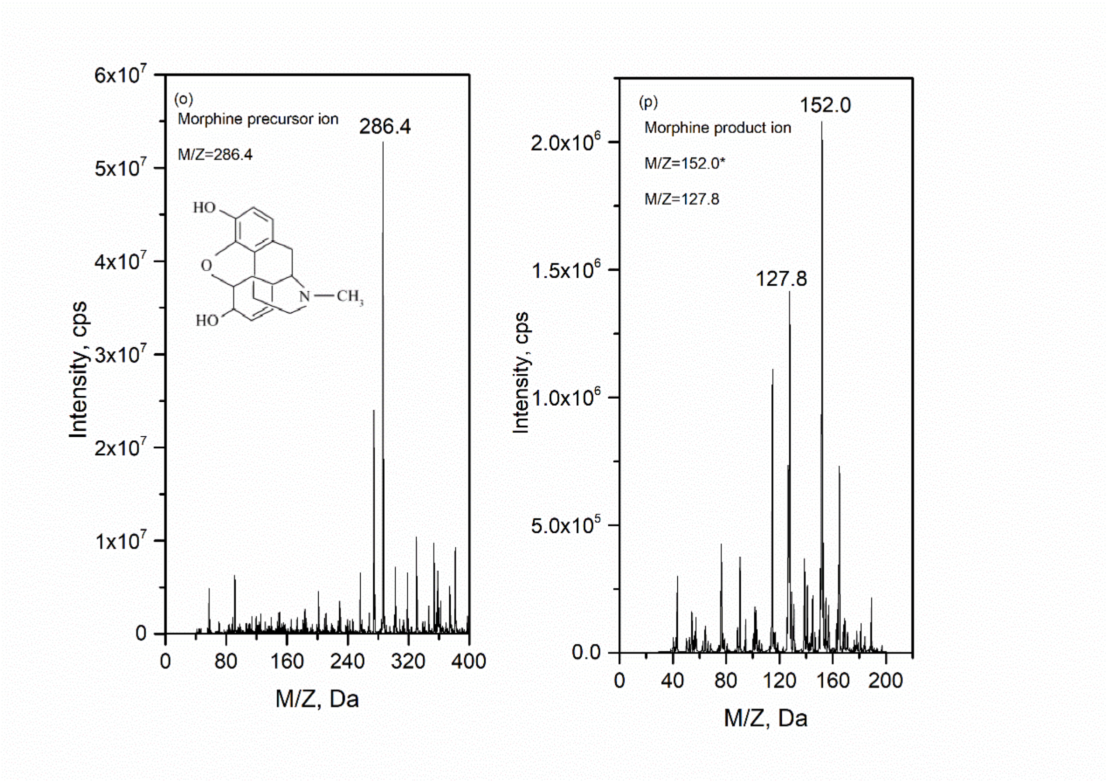
(o)
Morphine precursor ion
M/Z=286.4
286.4
HO
O
N—CH3
HO
Intensity, cps
M/Z, Da
(p)
Morphine product ion
M/Z=152.0*
M/Z=127.8
152.0
127.8
Intensity, cps
M/Z, Da

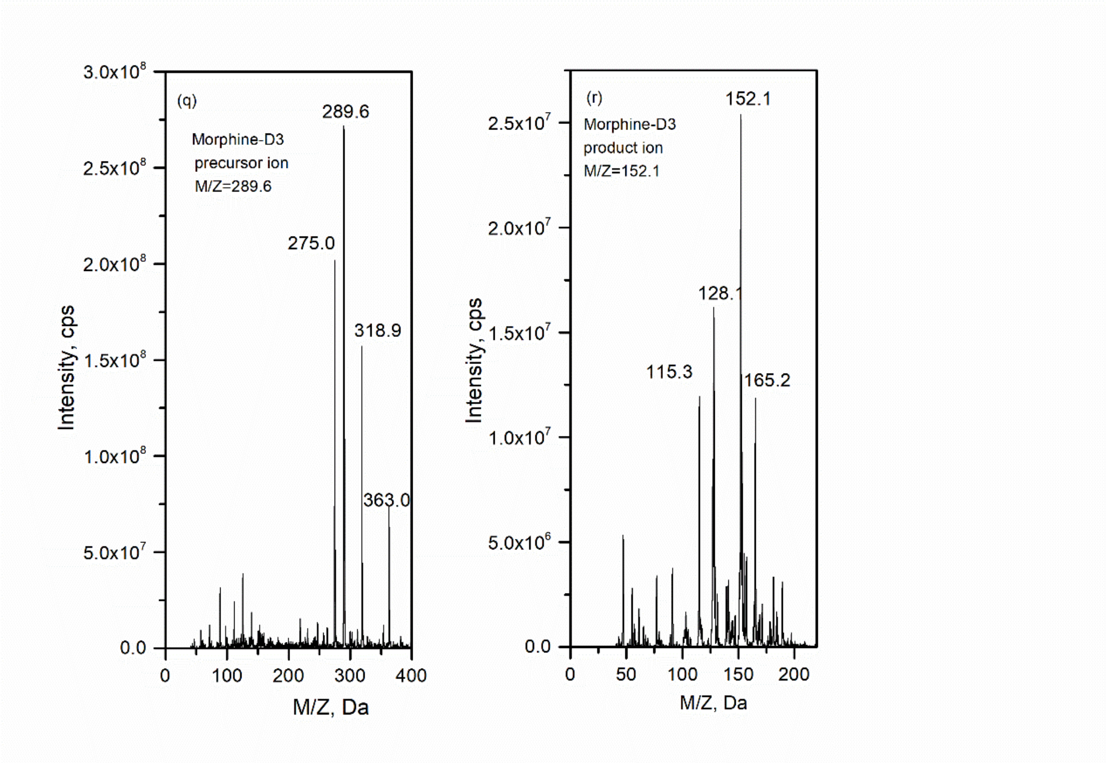
(q)
Morphine-D3
precursor ion
M/Z=289.6
289.6
275.0
318.9
363.0
Intensity, cps
M/Z, Da
(r)
Morphine-D3
product ion
M/Z=152.1
152.1
128.1
115.3
165.2
Intensity, cps
M/Z, Da

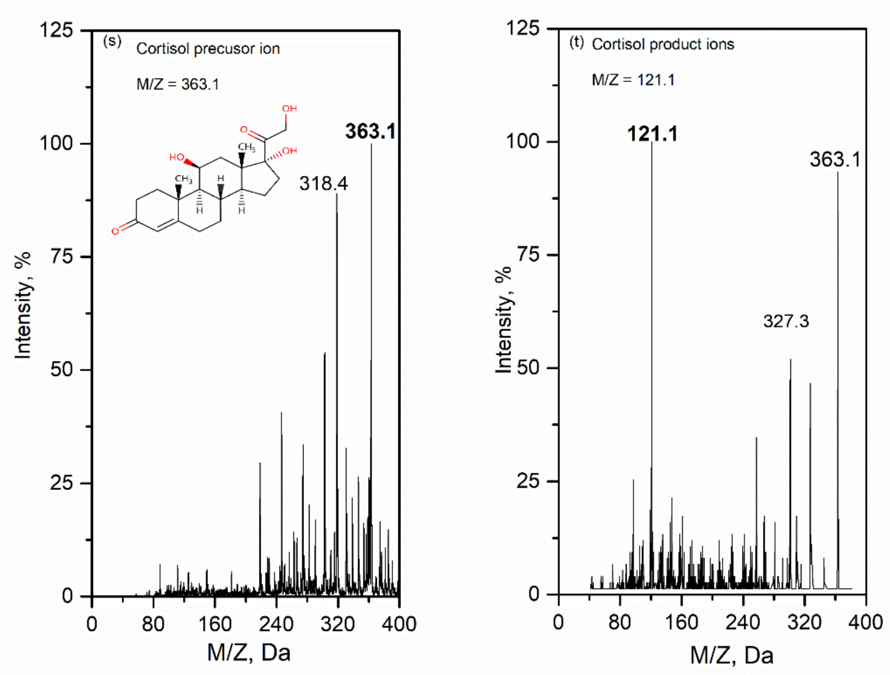
(s) Cortisol precusor ion
M/Z = 363.1
363.1
318.4
Intensity, %
M/Z, Da
(t) Cortisol product ions
M/Z = 121.1
121.1
363.1
327.3
Intensity, %
M/Z, Da

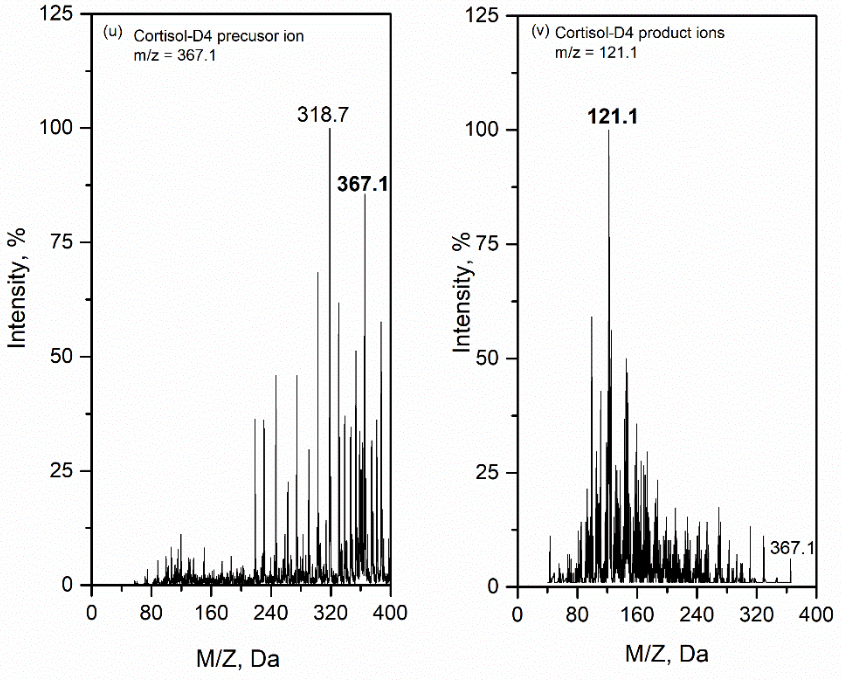
(u) Cortisol-D4 precusor ion
m/z = 367.1
318.7
367.1
Intensity, %
M/Z, Da
(v) Cortisol-D4 product ions
m/z = 121.1
121.1
367.1
Intensity, %
M/Z, Da

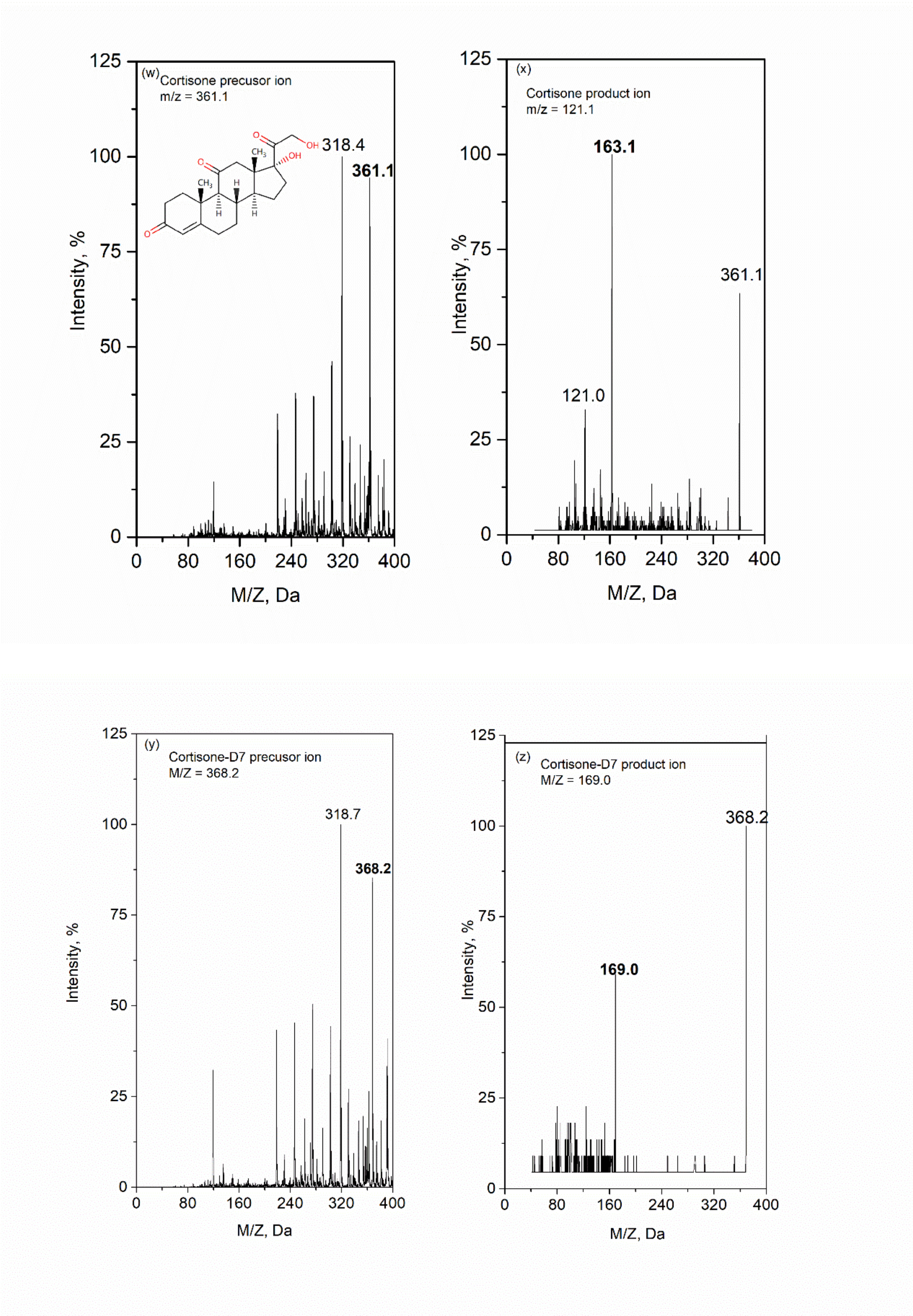

Figure 2. Optimized ion pairs of analytes, (a) MAM, methamphetamine precursor ion; (b) MAM product ion; (c) MAM-D5 precursor ion; (d) MAM-D5 product ion; (e) MDMA, 3,4-methylenedioxymethamphetamine precursor ion; (f) MDMA product ion; (h) ketamine precursor ion; (i) ketamine product ion; (j) ketamine-D4 precursor ion; (k) ketamine-D4 product ion; (l) heroin precursor ion; (m) heroin product ion; (n) 6-AM, 6-monoacetylmorphine precursor ion; (o) 6-AM, 6-monoacetylmorphine product ion; (p) morphine precursor ion; (q)morphine product ion; (r) morphine-D3 precursor ion; (s) morphine-D3 product ion; (t) cortisol precursor ion; (u) cortisol product ion; (v) cortisol-D4 precursor ion; (w) cortisol -D4 product ion; (x) cortisone precursor ion; (y) cortisone product ion; (a) cortisone-D7 precursor ion; (z) cortisone-D7 product ion; The determined

sensitivity for (a) ( ), (b) ( ), (c) ( ), (d) ( ), (e) (), and (f) ( )… spiked with and without 20 mg blank hair matrix. S means the sensitivity and R^2 is the square of correlation coefficient.
